# Enhancing Precision and Esthetics: Endoscopy-Assisted Total Maxillectomy for Locally Invasive Maxillary Ameloblastoma Using Contralateral Transmaxillary Approach Without Subciliary Incision

**DOI:** 10.7759/cureus.73192

**Published:** 2024-11-07

**Authors:** Masahiro Kikuchi, Eugene Nakamura, Seiji Oyagi, Shogo Shinohara, Norio Yamamoto

**Affiliations:** 1 Otolaryngology and Head and Neck Surgery, Kobe City Medical Center General Hospital, Kobe, JPN

**Keywords:** ameloblastoma, combined surgery, contralateral transmaxillary approach, endoscopic endonasal surgery, subciliary incision, total maxillectomy

## Abstract

This report presents a notable approach to treating a locally invasive maxillary ameloblastoma in a 46-year-old woman using an endoscopy-assisted total maxillectomy via a contralateral transmaxillary approach without a subciliary incision. Ameloblastomas, though benign, require radical surgical management due to their aggressive nature and high recurrence rates, especially in the maxilla. Traditional techniques often involve extensive facial incisions, leading to significant scarring and potential complications. In this case, the notable approach allowed for precise osteotomy of the pterygoid process and accurate delineation of the mucosal margins during ethmoidectomy while preserving facial esthetics. The surgery achieved complete tumor resection with successful reconstruction, avoiding the esthetic drawbacks associated with conventional methods. Postoperative recovery was uneventful, with no residual tumor observed at the six-month follow-up. This technique demonstrates the potential for reducing surgical morbidity and improving cosmetic outcomes in the management of complex maxillary tumors.

## Introduction

Ameloblastomas are benign odontogenic tumors characterized by local invasiveness and a high recurrence rate, necessitating a comprehensive surgical intervention to ensure complete removal and minimize the recurrence risk. These tumors more commonly arise in the mandible than in the maxilla. Maxillary ameloblastomas exhibit unique genetic mutations, such as SMO and RAS mutations, and require a more radical surgical treatment than mandibular ameloblastomas to reduce the recurrence rates [1]. Additionally, although the malignant transformation rate of ameloblastomas is approximately 3%, the risk is higher for maxillary than for mandibular tumors [2]. Due to their aggressive nature, standard management strategies for maxillary ameloblastomas commonly involve en-bloc resection with adequate bone margins, including total maxillectomy, often necessitating reconstruction to restore function and esthetics [1,3]. The Weber-Ferguson incision with subciliary extension is commonly used in total maxillectomy for malignant maxillary tumors to access the posterior maxilla and perform pterygoid process osteotomy. While it offers a broad visual field, it also has drawbacks, such as large facial scars and potential lower eyelid malposition due to scarring from the subciliary incision.

Herein, we present a case of locally invasive maxillary ameloblastoma in a 46-year-old woman who was treated by combined surgery using the contralateral transmaxillary approach [4-6] without subciliary incision. This notable approach allowed for precise osteotomy of the pterygoid process from the middle cranial base, accurate determination of the osteotomy line for the maxillary frontal process from within the nasal cavity, and precise and sufficient mucosal margin delineation during ethmoidectomy, reducing the risk for residual disease while preserving facial esthetics.

## Case presentation

**Figure 1:**
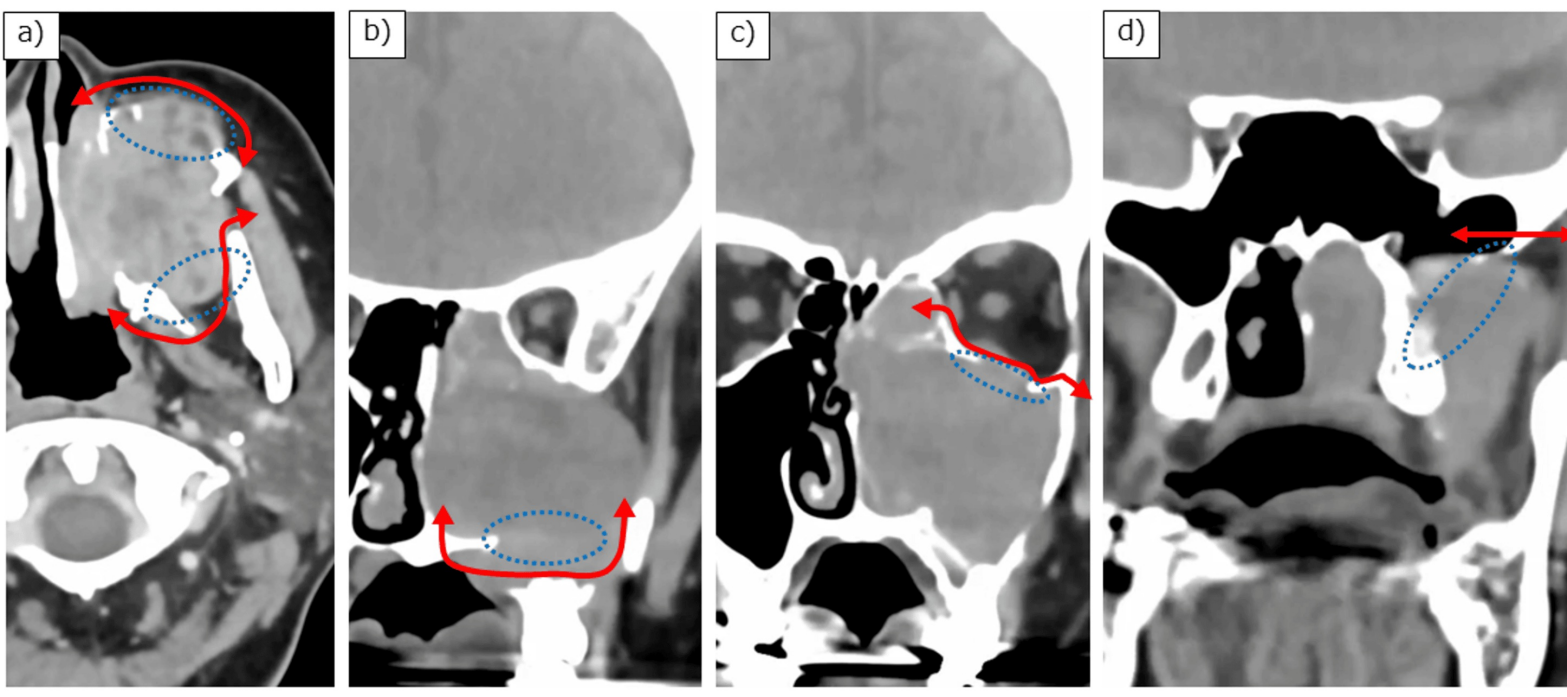
Preoperative computed tomography images. (a) Axial image showing bone infiltration in the anterior and posterior maxillary walls; (b, c) Coronal images showing infiltration in the inferior and superior maxillary walls, respectively; and (d) The base of the sphenoid pterygoid process. Blue dotted line: bone infiltration site; red arrow: resection line.

**Figure 2:**
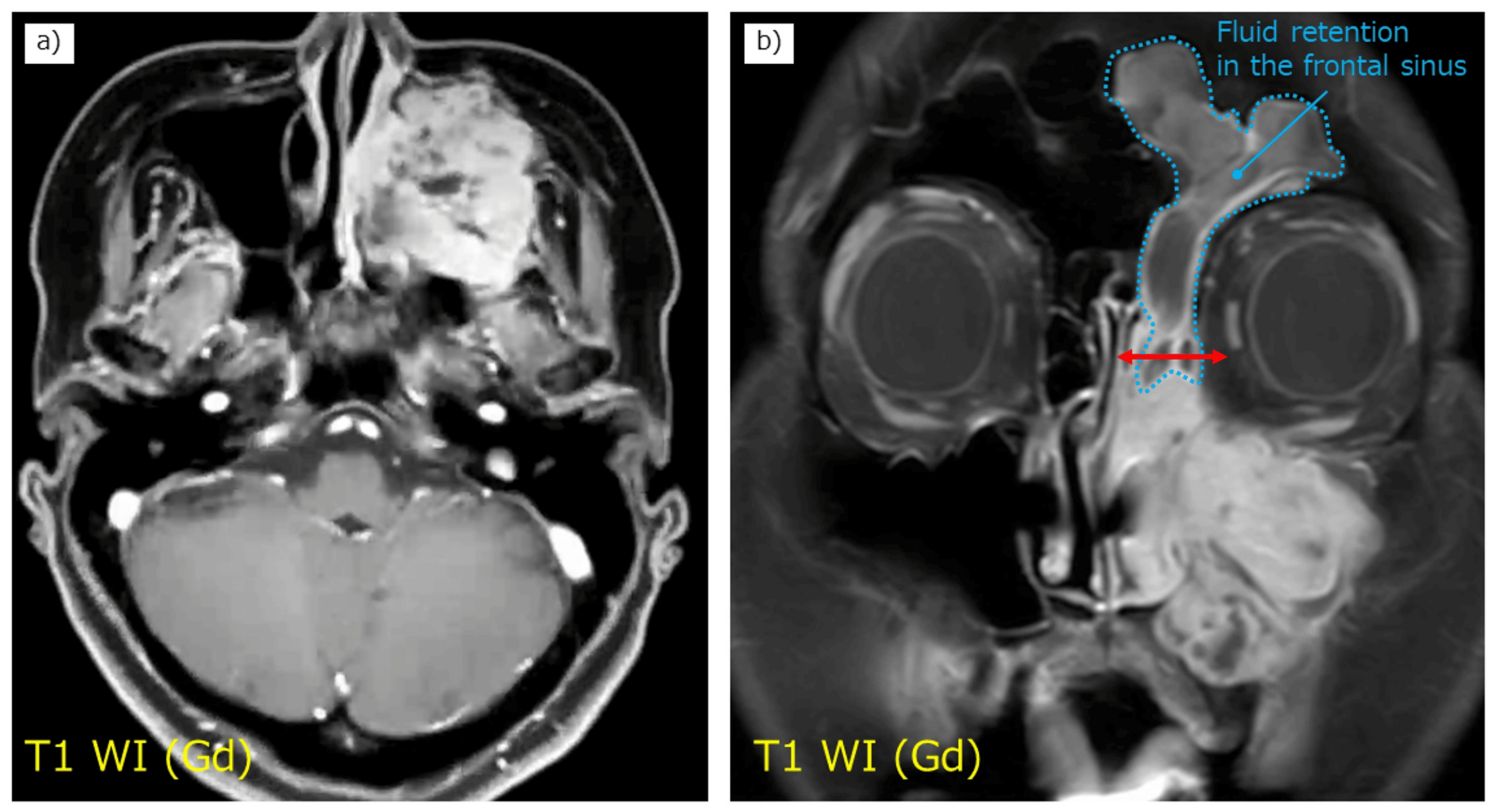
Preoperative contrast-enhanced magnetic resonance imaging scans. (a) Axial and (b) coronal T1-weighted images showing fluid accumulation (blue dotted line) in the frontal sinus without tumor infiltration. The images indicate that the osteotomy in the anterior, medial, and superior directions can be performed at the level of the ethmoid sinus, as indicated by the red line.

A 46-year-old woman with no significant past medical history presented with a chief complaint of left nasal obstruction. On physical examination, there was evident swelling of the left cheek area. Fiberoptic nasopharyngoscopy revealed a mass in the left middle nasal meatus, which was firm and nontender, with no signs of ulceration or discharge. Computed tomography (CT) of the head and neck showed a tumor centered in the left maxillary sinus with extensive bone infiltration (Figure 1), as well as soft tissue density in the left frontal sinus. The tumor extended medially into the nasal cavity, laterally into the infratemporal fossa, posteriorly into the pterygoid plate, anteriorly into the facial subcutaneous tissue, and superiorly into the orbit. Magnetic resonance imaging (MRI) confirmed the localization of the tumor and fluid retention without tumor invasion in the left frontal sinus (Figure 2). No tumor invasion was observed in the sphenoid sinus on either CT (Figure 1d) or MRI.

Based on the clinical examination and imaging findings, a biopsy was performed of the tumor mass extending into the left middle nasal meatus. The histopathological examination confirmed the diagnosis of ameloblastoma, and the patient was scheduled for surgical treatment.

Surgical procedure

**Figure 3:**
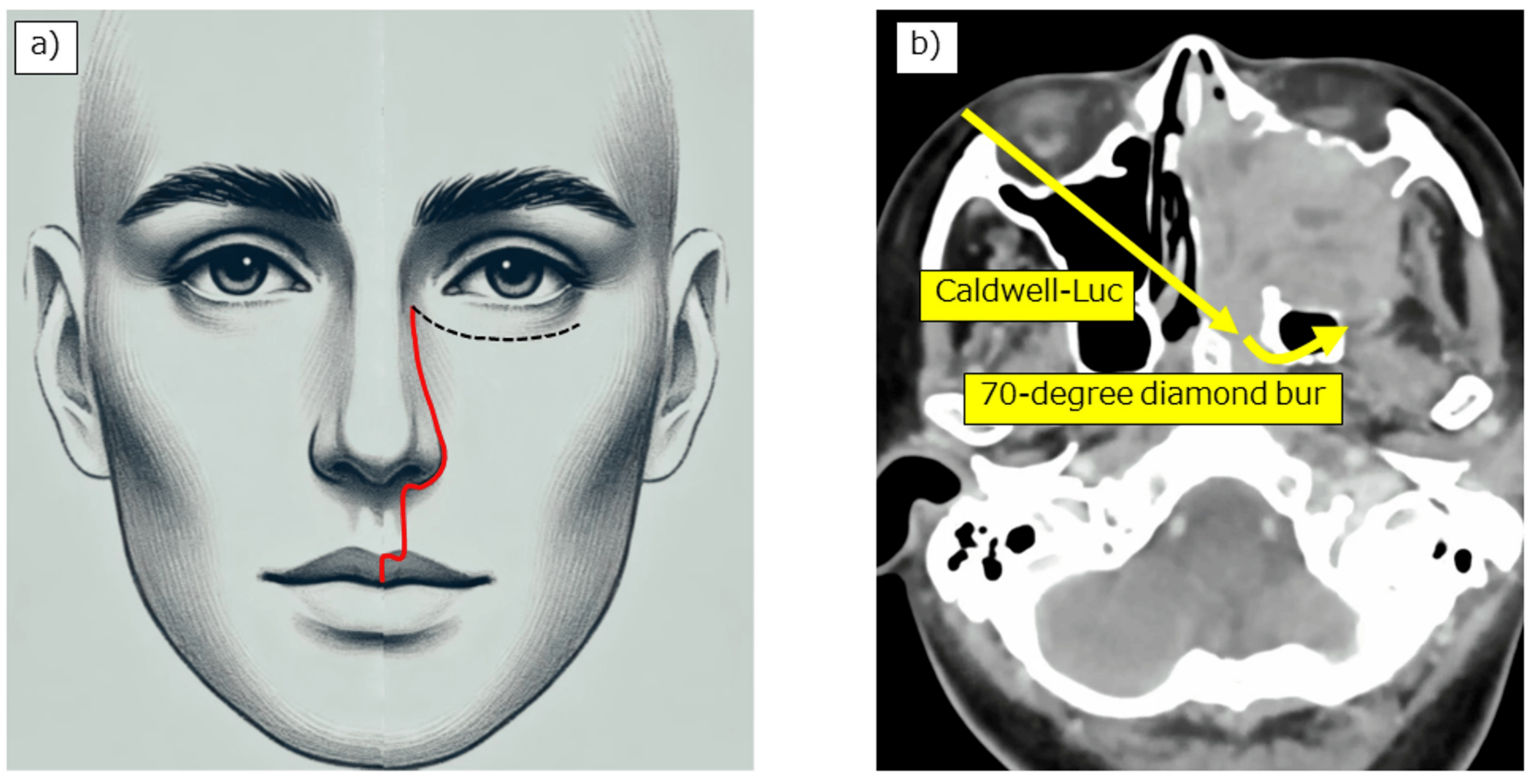
Two notable aspects in this case. (a) Considering esthetics, the Weber-Ferguson incision was performed without subciliary extension. (b) Since this facial incision line does not allow for a lateral approach, the contralateral maxillary sinus was opened using the Caldwell-Luc procedure, and a 70-degree bur was inserted to drill the base of the pterygoid process within the sphenoid sinus using the contralateral transmaxillary approach. Note: The facial illustration in this figure was created using AI tools specifically for this publication.

We performed combined surgery using the contralateral transmaxillary approach without a subciliary incision (Figure 3). The surgical procedure is visualized in Video 1.

**Video 1:** Endoscopy-assisted total maxillectomy via a contralateral transmaxillary approach without subciliary incision for a locally invasive maxillary ameloblastoma. Combined surgery using the contralateral transmaxillary approach without subciliary incision was performed. This detailed step-by-step procedure illustrates the less invasive nature of the approach, preserving facial esthetics while ensuring complete tumor resection.

Through the right nasal cavity, we drilled the vomer and the anterior wall and septum of the sphenoid sinus. After identifying the bony prominences of the Vidian canal and V2 (maxillary nerve) at the floor and upper lateral part of the left sphenoid sinus, respectively, we initially attempted to drill the floor of the left sphenoid sinus using a 15-degree diamond bur through the left nasal cavity. However, this approach did not allow sufficient reach to the lateral side of the Vidian canal due to angle limitations. Therefore, we proceeded with a contralateral transmaxillary approach from the right side to obtain the appropriate trajectory.

We fractured the right uncinate process at the transition between the vertical and horizontal parts and removed both parts to enlarge the ostium of the right maxillary sinus. We also excised the lower half of the right middle turbinate and flipped the remaining middle turbinate upwards. Next, the Caldwell-Luc procedure was performed on the right side, removing the anterior wall of the maxillary sinus with a chisel and hammer to preserve the infraorbital nerve. We then inserted a 70-degree diamond bur (30K RTdiamond bur 4.0 mm 70°; Medtronic, Minneapolis, USA) through the anterior wall of the right maxillary sinus, passed it through the right maxillary sinus into the left sphenoid sinus, reaching lateral to the left Vidian canal, and cut the bone from the floor to the lateral wall of the sphenoid sinus cavity, detaching the base of the left pterygoid process from the middle cranial base.

Subsequently, the left lateral nasal wall was incised vertically at the anterior end of the left inferior turbinate, and the left maxillary frontal process was osteotomized at the level of the agger nasi, exposing the subcutaneous tissue. We then opened the left agger nasi cell, opened a drainage route to the left frontal sinus, and drained the collected pus. Next, we cut the left uncinate process cranially, identifying and preserving the medial orbital wall. After identifying the ethmoid roof and anterior ethmoidal artery, we gathered the ethmoid sinus mucosa in the medial and inferior areas and performed ethmoidectomy. The left middle turbinate was cut at the skull base level.

**Figure 4:**
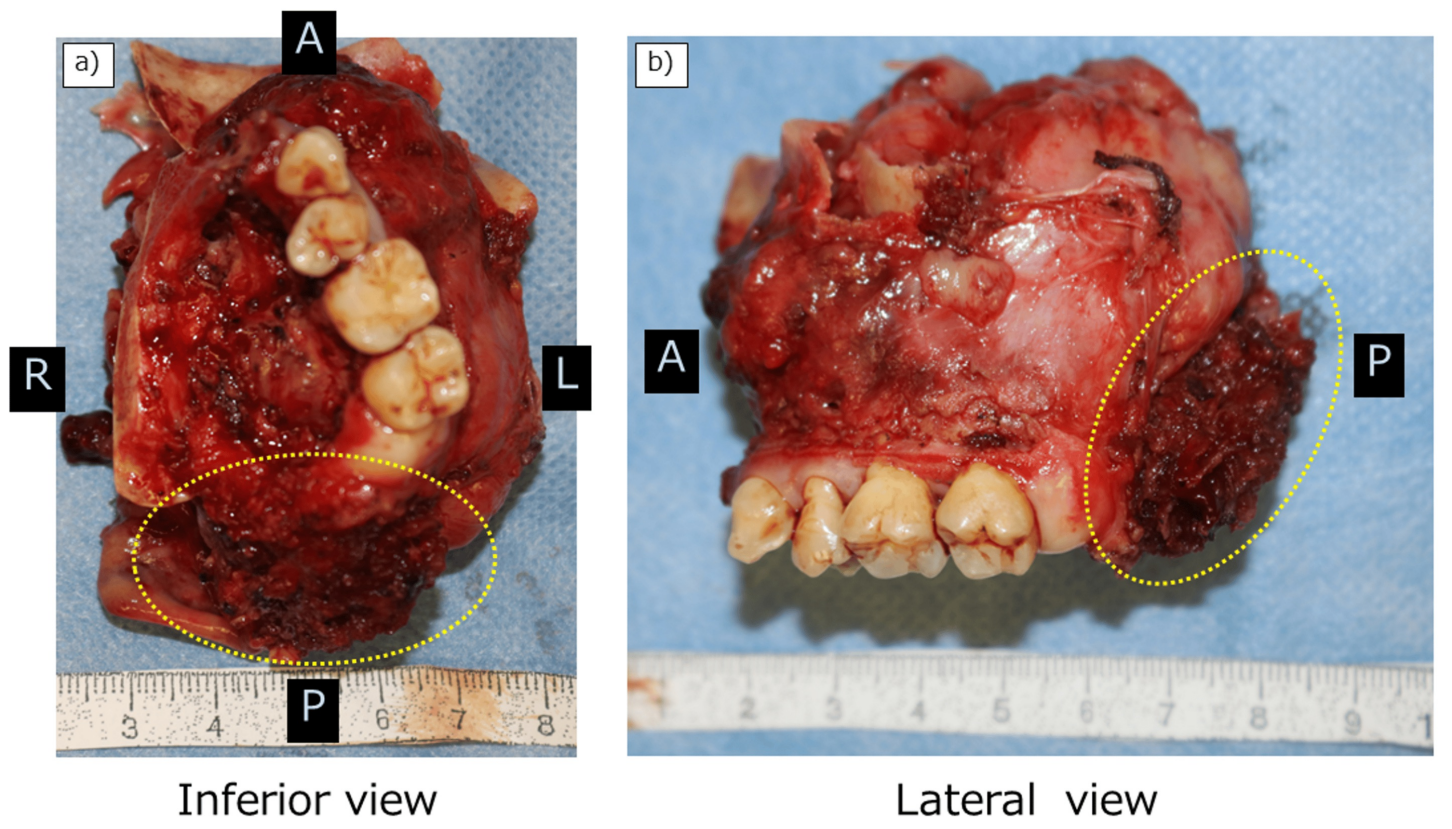
Resected specimen. (a) Inferior and (b) lateral views. Note that the pterygoid process and lateral pterygoid muscle are adequately resected (yellow dotted line).

We then applied the Weber-Ferguson facial incision without subciliary extension. The facial flap was elevated without cutting into the tumor. The pre-osteotomized maxillary frontal process area from within the nasal cavity was confirmed, and the osteotomy line was extended to the infraorbital wall. We performed endoscopy-assisted subperiosteal dissection of the infraorbital wall. After identifying the lower orbital fissure, we inserted a wire saw and performed an osteotomy from the zygoma. We then extracted the left second premolar and performed an osteotomy of the hard palate, thus completing the osteotomy procedure. The palatal mucosa was resected together with the tumor. The surrounding muscles (pterygoid muscles) and soft tissue were cut using a LigaSure Exact Dissector (Covidien Japan, Tokyo, Japan), and the tumor was resected en bloc (Figure 4).

The orbital floor was reconstructed using SuperFIXSORB (Teijin Medical Technologies Co., Ltd., Tokyo, Japan), and the defect in the maxilla and palate was reconstructed with a free rectus abdominis myocutaneous flap.

Postoperatively, the patient recovered well. At the six-month follow-up, the surgical site showed satisfactory healing. The patient reported significant improvement in nasal breathing and facial appearance. Imaging studies confirmed the absence of residual tumors and proper placement of the reconstructive materials.

This case report was conducted under the approval of the Institutional Review Board of Kobe City Medical Center General Hospital (approval number: 24145). This protocol is disclosed on our hospital’s website, providing patients the option to opt out of participation. Therefore, specific written informed consent was not required for this case.

## Discussion

This case highlights the notable use of endoscopic techniques, which allowed for precise osteotomy of the pterygoid process from the middle cranial base, accurate determination of the osteotomy line for the maxillary frontal process from within the nasal cavity, and precise and sufficient mucosal margin delineation during ethmoidectomy, thereby enhancing the overall surgical outcome and minimizing facial incisions.

Our surgical approach has two notable aspects. The first aspect is the successful use of the contralateral transmaxillary approach without subciliary incision to resect the pterygoid process from the skull base. The Weber-Ferguson incision with subciliary extension has been routinely used to expose the posterior aspect of the maxilla and perform pterygoid process osteotomy during total maxillectomy for malignant maxillary tumors. Although this facial incision technique has the advantage of providing a wide visual field, it also has associated disadvantages, including an esthetically unpleasant large facial incision and possible lower eyelid malpositioning due to postoperative scar formation in the subciliary incision area. In contrast, our approach allowed for a less invasive surgery by avoiding the subciliary incision and minimized the risk for postoperative complications, such as lower eyelid malpositioning, enhancing the overall esthetic outcome for the patient [7]. The contralateral transmaxillary technique involved using a 70-degree diamond bur inserted through the contralateral maxillary sinus to precisely osteotomize the base of the pterygoid process from the skull base. This method provided excellent visualization and access to the pterygoid process from the medial side without cutting into the tumor on the lateral side, significantly reducing the risk for recurrence. Hanazawa et al. also used the contralateral transmaxillary approach for osteotomy of the pterygoid process [4]. However, they performed a subciliary incision for facial access, which can lead to lower eyelid malpositioning.

The second notable aspect is the effective application of endoscopic techniques during ethmoidectomy to achieve accurate determination of the osteotomy line for the maxillary frontal process and safe mucosal margin delineation. In total maxillectomy, maxillary frontal process osteotomy is usually performed through an external facial incision, but this technique does not allow for completing the osteotomy with confidence at the correct height in the ethmoid sinus. However, if the incision is made from within the nasal cavity using our method, the osteotomy can always be performed at the correct level. The osteotomy line can be easily identified at the time of subsequent elevation of the facial flap and connected outward. Endoscopic ethmoidectomy provided clear visualization, allowing for safe and accurate removal of the tumor [8,9]. Employing these techniques, particularly for maxillary sinus tumors extending into the ethmoid sinus, can minimize the risk of recurrence and improve the overall prognosis of patients.

The present case enriches the knowledge of the surgical management of locally invasive maxillary ameloblastoma by demonstrating a notable surgical approach that minimizes facial incisions and ensures thorough tumor removal. Additionally, the combined endoscopic and external approach used in this case can be applied in the treatment of maxillary sinus cancer, demonstrating its versatility and potential in managing other complex maxillofacial tumors. Nagaoka et al. reported on the advantages of using an endoscopy-assisted approach for total maxillectomy, highlighting the ability to achieve precise surgical margins, particularly in the posterior region, which is often difficult due to its deep location and proximity to the pterygoid venous plexus [10]. Their technique allowed for a more accurate resection of the pterygoid process, enhancing the overall precision of the surgery without extending the operation time.

## Conclusions

In conclusion, we presented a combined approach for performing total maxillectomy without subciliary incision using an endoscopy-assisted contralateral transmaxillary approach and ethmoidectomy for locally invasive maxillary ameloblastoma. This adaptation, combining established techniques, enabled precise osteotomy and mucosal margin delineation, potentially reducing the risk of residual disease while preserving facial esthetics in a young patient. While the esthetic and surgical access advantages are case-dependent and modest, this combined approach may offer an alternative option in selected cases of maxillary ameloblastomas and similar complex maxillofacial tumors. Further studies are needed to evaluate the broader applicability and long-term outcomes of this technique.

## References

[1] Evangelou Z, Zarachi A, Dumollard JM, Peoc'h M, Komnos I, Kastanioudakis I, Karpathiou G (2020). Maxillary ameloblastoma: a review with clinical, histological and prognostic data of a rare tumor. In Vivo.

[2] Liu W, Zheng C, Zhang X, Hu H (2024). Recurrence and malignant risk of ameloblastoma: a demographic study of 1626 cases from east China. Oral Oncol.

[3] Armocida D, Berra LV, Pucci R, Battisti A, Della Monaca M, Valentini V, Santoro A (2022). Ameloblastoma and intracranial involvement: the current challenge of the radical surgical treatment. Comprehensive review of the literature and institution experience. J Maxillofac Oral Surg.

[4] Hanazawa T, Yamasaki K, Chazono H, Okamoto Y (2018). Endoscopic contralateral transmaxillary approach for pterygoid process osteotomy in total maxillectomy: a technical case report. Auris Nasus Larynx.

[5] Pamias-Portalatin E, Mahato D, Rincon-Torroella J, Vivas-Buitrago T, Quiñones-Hinojosa A, Boahene KO (2019). Endoscope-assisted contralateral transmaxillary approach to the clivus and the hypoglossal canal: technical case report. J Neurosurg.

[6] Porto E, Vuncannon J, Revuelta Barbero JM (2023). Contralateral Transmaxillary approach to petrous apex granuloma with lateral Maxillotomy: 2-dimensional operative video. World Neurosurg.

[7] Kim CH, Choi WY, Son KM, Cheon JS (2020). Prediction of lower eyelid malpositioning after surgical correction of orbital fracture using the Subciliary approach through the canthal area and orbital vector analysis. J Craniofac Surg.

[8] Carta F, Kania R, Sauvaget E, Bresson D, George B, Herman P (2011). Endoscopy skull-base resection for ethmoid adenocarcinoma and olfactory neuroblastoma. Rhinology.

[9] Rawal RB, Gore MR, Harvey RJ, Zanation AM (2012). Evidence-based practice: endoscopic skull base resection for malignancy. Otolaryngol Clin North Am.

[10] Nagaoka M, Omura K, Nomura K, Takeda T, Otori N, Kojima H (2023). Endoscopic-assisted total maxillectomy with precise surgical margins. Head Neck.

